# Supplementary material for: The Effect of Climate Change on Food Crop Production in Lagos State
Source: Foods. 2022 Dec 9;11(24):3987. doi: 10.3390/foods11243987 (PMC9778574; doi:10.3390/foods11243987)
Supplement: Supplementary file 1 [file foods-11-03987-s001.zip › foods-2036822-supplementary.pdf]

LAGOS STATE UNIVERSITY, OJO
FACULTY OF SOCIAL SCIENCES
DEPARTMENT OF GEOGRAPHY AND PLANNING
SURVEY QUESTIONNAIRE

Dear Sir/Ma,

RESEARCH QUESTIONNAIRE ON THE PERCEPTION OF FARMERS ON THE EFFECT OF CLIMATE CHANGE ON FOOD CROP PRODUCTION

I am an undergraduate of the above named institution. I am conducting a research on the topic titled "Effect of Climate Change on Food Crop Production in Lagos state. You are requested to assist answer/complete the questions as it relates to your activities. This survey is purely for academic purpose and all information supplied will be treated confidentially.

PART A: (Demographic data)

1. **Location** [a] Epe [b] Ikorodu [c] Badagry
2. **Sex:** Female ( ) Male ( )
3. **Age:** 20 < ( ) 21 – 30 ( ) 31 – 40 ( ) 41 – 50 ( ) 51 and above ( )
4. **Income**: Below 20,000( ) 21000 – 50000 ( ) 51000 – 80000 ( ) 81000 – 100000 ( )
5. **Occupation:** Civil Servant ( ) Artisan ( ) Unemployed ( ) Retired ( ) Self Employed ( ) farming ( ) Others ( )
6. **Marital status:** Single ( ) Married ( ) Divorced ( ) Widow/Widower ( )
7. **House hold size:** 1-5 ( ) 6-10 ( ) 10-15 ( ) Others ( )
8. **Level of education:** No Formal Education ( ) Primary ( ) Secondary ( ) Tertiary ( )
9. **Ethnicity:** Igbo ( ) Hausa ( ) Yoruba ( ) Others ( )
10. **Religion:** Christianity ( ) Islam ( ) traditional ( )
11. **Do you own a farmland?** Yes ( ) No ( )
12. **If yes, what is the average size of your farmland?** [a] 1 – 5 plots [b] 1 – 5 acres [c] 1 – 5 Hectares
13. **How long have you been farming?** [a] 1 – 5 years [b] 6 – 10 years [c] 11 – 15 years [d] 16 years and above

PART B: (Awareness)

14. Have you heard of climate change? Yes ( ) No ( ) don't know ( )
15. If yes, how/where? (a) Television/Radio (b) Friends (c) Convention/Programmes (d) Researchers (e) farmers cooperatives (f) extension workers (g) others
16. Have you ever experience excessive rainfall, erosion and flooding? Yes ( ) No ( ) don't know ( )
17. If yes, are you aware that this is an evidence of climate change? Yes ( ) No ( ) don't know ( )
18. What was its effect on your crop production? Good ( ) bad ( ) fair ( )
19. Are you aware that climate change can affect food crop production? Yes ( ) no ( ) don't know ( )
20. Do you have challenges with Pest and Diseases? Yes ( ) No ( )
21. If yes? What period of time? ________ (a) Raining (b) Dry (c) Harmattan
22. Do you know this is one of the effects of climate change? [a] Yes [b] No
23. **What type of crop do you plant? Cassava ( ) Maize ( ) Vegetable ( )**

Effect of Climate Change on Food Crop Production

24. Do you believe climate change can affect the output of your crop? Yes ( ) No ( ) Don't know ( )
25. How will you rate your rate of crop yield/output in the last 3 decades? (a) Bad, (b) Fair, (c) Good
26. Do you engage in some of these activities?

S/N	ACTIVITIES	YES	NO
a.	Bush burning		
b.	Deforestation		
C	Burning of woodfuel		
d.	Use of fertilizer		
e.	Use of chemicals like pesticides, herbicides etc		
f.	Continuous cropping		
g.	Over grazing		

27. Are you aware that some of these activities can lead to climate change? Yes ( ) No ( ) don't know ( )
28. Do you know that this can reduce the rate of crop production output? Yes ( ) No ( ) don't know ( )
29. How will you describe the pattern and weather condition of the following?

S/N	VARIABLES/SEASONS	INCREASING	DECREASING	NO CHANGES
a.	Rainfall			
b.	Long period of dry season			
c.	Harmattan			
d.	Temperature			
e.	Thunderstorms			
f.	Heavy Winds			
g.	Floods			
h.	Drought			
i.	Heat Waves			
j.	Desertification			
k.	Loss Of Forest Resources			
l.	Soil erosion			
m.	Soil moisture			
n.	Pests			
0.	Diseases			
p.	Weeds			

30. What is the extent to which the perception of the Famers on the Weather conditions affects crop yield?

S/N	VARIABLES/SEASONS	High Positive	Low Positive	High Negative	High Negative
a.	Rainfall				
b.	Long period of dry season				
c.	Harmattan				
d.	Temperature				
e.	Thunderstorms				
f.	Heavy Winds				

g.	Floods				
h.	Drought				
i.	Heat Waves				
j.	Desertification				
k.	Loss of Forest Resources				
l.	Soil erosion				
m.	Soil moisture				
n.	Pests				
0.	Diseases				
p.	Weeds				

31. What are the coping strategies adopted by you to manage climate change situation?

S/N	COPING STRATEGIES	YES	NO
a.	Multiple/intercropping		
b.	Agro-forestry/aforestation,		
c.	Purchase/harvest of water for irrigation		
d.	Mulching		
e.	Use of resistant varieties.		
f.	Expansion of cultivated land area		
g.	Use of chemical: herbicides, pesticides, etc.		
h.	Increased used of fertilizers		
i.	Intensive manure application		
j.	Others		
